# Osteoinductive potential of graphene and graphene oxide for bone tissue engineering: a comparative study

**DOI:** 10.1186/s13018-024-05028-9

**Published:** 2024-08-30

**Authors:** Shivaji Bhikaji Kashte, Sachin Kadam, Nicola Maffulli, Anish G. Potty, Filippo Migliorini, Ashim Gupta

**Affiliations:** 1 Department of Stem Cell and Regenerative Medicine, Centre for Interdisciplinary Research, D. Y. Patil Education Society (Institution Deemed to be University), Kolhapur, 416006 India; 2 Sophisticated Analytical and Technical Help Institute, Indian Institute of Technology Delhi, Hauz Khas, New Delhi, 110016 India; 3 Department of Trauma and Orthopaedic Surgery, Faculty of Medicine and Psychology, University La Sapienza, 00185 Rome, Italy; 4 Barts and the London School of Medicine and Dentistry, Centre for Sports and Exercise Medicine, Queen Mary University of London, London, E1 4DG UK; 5 School of Pharmacy and Bioengineering, Keele University School of Medicine, Stoke-on-Trent, ST5 5BG UK; 6 South Texas Orthopaedic Research Institute (STORI Inc.), Laredo, TX 78045 USA; 7 Department of Orthopaedic, Trauma, and Reconstructive Surgery, RWTH University Medical Centre, Pauwelsstraße 30, 52074 Aachen, Germany; 8 Department of Life Sciences, Health, and Health Professions, Link Campus University, Rome, Italy; 9 Department of Orthopaedic and Trauma Surgery, Academic Hospital of Bolzano, Bolzano Italy; 10 Future Biologics, Lawrenceville, GA 30043 USA

**Keywords:** Osteoinductivity, Graphene, Umbilical cord, Osteogenic differentiation, Scaffolds, Bone defects, Bone tissue engineering

## Abstract

**Background:**

Bone defects, especially critical-size bone defects, and their repair pose a treatment challenge. Osteoinductive scaffolds have gained importance given their potential in bone tissue engineering applications.

**Methods:**

Polycaprolactone (PCL) scaffolds are used for their morphological, physical, cell-compatible and osteoinductive properties. The PCL scaffolds were prepared by electrospinning, and the surface was modified by layer-by-layer deposition using either graphene or graphene oxide.

**Results:**

Graphene oxide-coated PCL (PCL-GO) scaffolds showed a trend for enhanced physical properties such as fibre diameter, wettability and mechanical properties, yield strength, and tensile strength, compared to graphene-modified PCL scaffolds (PCL-GP). However, the surface roughness of PCL-GP scaffolds showed a higher trend than PCL-GO scaffolds. In vitro studies showed that both scaffolds were cell-compatible. Graphene oxide on PCL scaffold showed a trend for enhanced osteogenic differentiation of human umbilical cord Wharton’s jelly-derived Mesenchymal Stem Cells without any differentiation media than graphene on PCL scaffolds after 21 days.

**Conclusion:**

Graphene oxide showed a trend for higher mineralisation, but this trend is not statistically significant. Therefore, graphene and graphene oxide have the potential for bone regeneration and tissue engineering applications. Future in vivo studies and clinical trials are warranted to justify their ultimate clinical use.

**Graphical abstract:**

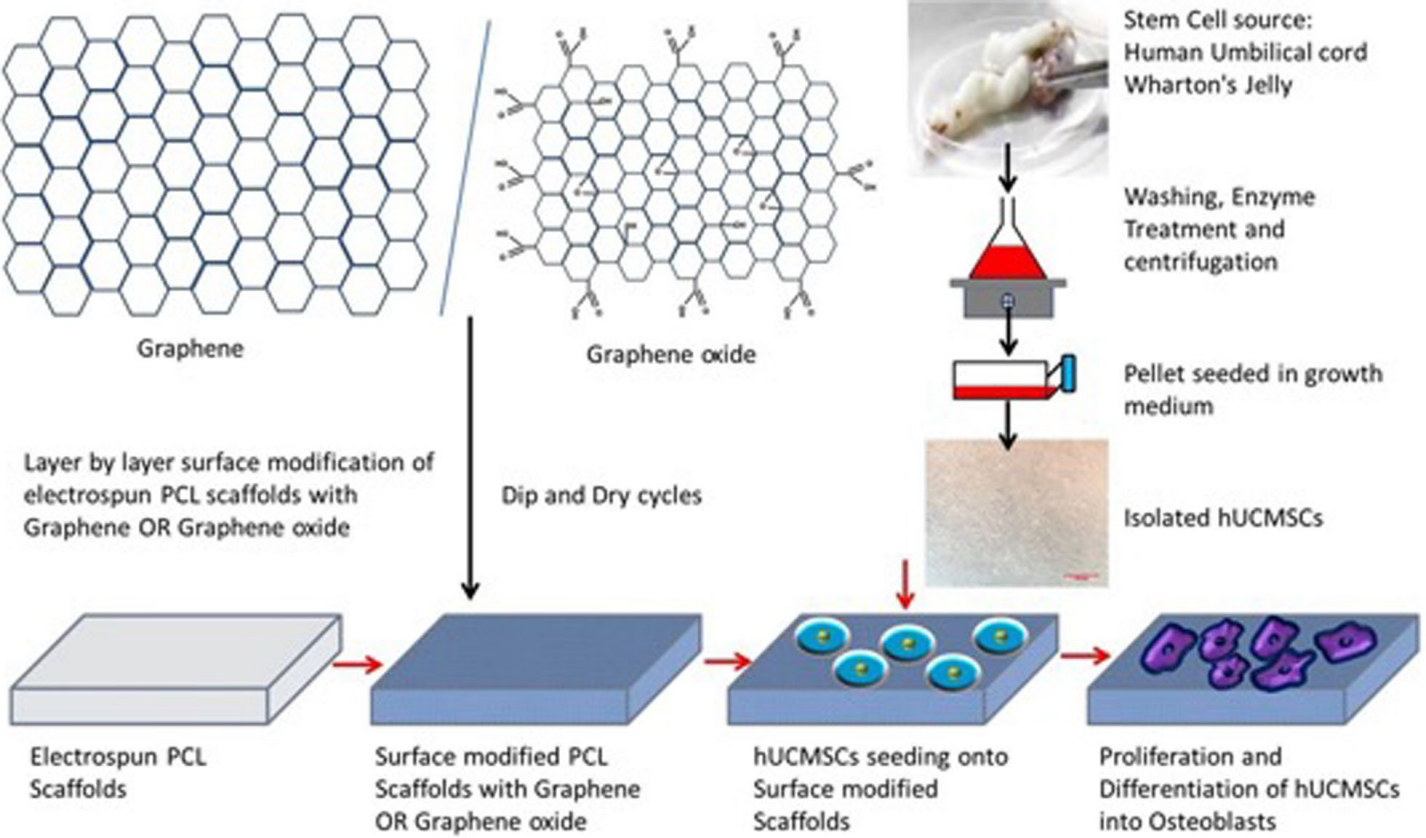

## Introduction

Bone defects, especially critical size bone defects, pose a major clinical challenge [1–3]. Bone is the second most commonly transplanted tissue after blood, and more than 2.2 million bone graft procedures are performed worldwide every year. Autografts and allografts are widely used for bone tissue regeneration, but are expensive and associated with low tissue availability, disease transmission, and tissue morbidity [1–4].

Bone tissue engineering employs cells, scaffolds, and growth factors in novel fashions to optimise regeneration. Scaffolds provide anatomical support to the mesenchymal stem cells for their attachment, migration, and proliferation. They are able to differentiate into the osteoblasts, the bone-forming cells that repair damaged bone tissues [5, 6]. These scaffolds should be biocompatible, biodegradable, osteoconductive and have high mechanical strength [7, 8]. However, the osteoinductivity of scaffolds limits the healing of large bone defects. Therefore, healing promotive factors or growth factors such as vascular endothelial growth factor (VEGF), transforming growth factor- β (TGF-β), platelet-derived growth factor (PDGF), and bone morphogenetic proteins (BMPs), e.g., BMP-2, BMP-7 and insulin-like growth factor (IGF) [3, 8] modulating osteoinduction have been incorporated into scaffolds to enhance their osteoinductivity [9, 10]. Osteoinductive materials stimulate the repair of tissues in the areas which, if left untreated, cannot heal on their own [11]. Therefore. osteoinductive scaffolds have great potential for bone tissue engineering.

Graphene (GP) and its derivatives graphene oxide (GO) are a “wonder material” given their biomechanical properties. Their excellent potential for regenerating bone tissue is associated with high mechanical strength, electrical conductivity, large surface area, atomic structure stability, and the ability to promote cell proliferation and differentiation [12, 13]. The primary issue with the failure of implants is osseointegration. GP and its derivatives can be coated onto the surface of implants to enhance osteointegration and bone formation [13]. GP is a synthetic atomic layer of graphite with SP2-bonded carbon atoms arranged in a honeycomb lattice structure. GP, a 2D structure, is where every single atom is exposed to a chemical reaction from either side [14]. GO, obtained from the oxidation of GP, contains epoxy and carbon radicals in the basal planes, with carboxyl and hydroxyl groups on its edges [15]. Also, GO has more hydrophilic groups and easy dispersion ability [16]. GP and GO are potential candidates for surface modification of scaffolds. Surface modification of scaffolds enhances surface charge, wettability, roughness or topography and ultimately, cellular attachment and proliferation [16, 17].

The layer-by-layer deposition method is a simple, relatively fast, environmentally friendly, and low-cost process [18] with controllable deposit thickness and uniform surface coverage [19]. Layer-by-layer deposition significantly enhances surface properties such as hydrophilic nature and mechanical strength of scaffolds [18] and enhances cell viability and cell attachment of epidermal cells [19–21]. GO-poly-l-lysine composites [22], GO with Polylactic acid (PLA) and hydroxyapatite (HA) [23], GO along with HA and chitosan functionalised GP nano-sheets covered with polyvinyl alcohol [24], GP and GO on polydimethylsiloxane (PDMS) [25], GO doped poly (lactic-co-glycolic acid) (PLGA) scaffolds [26], have been studied for bone regeneration applications. GP and GO and their interaction with stem cells revealed cellular compatibility and the ability to support the differentiation of stem cells into osteoblasts, chondroblasts and neuronal lineages [27–29].

In a previous study, layer-by-layer deposition of GP and GO onto electrospun PCL scaffolds improved hydrophilicity, cell compatibility and osteoinductivity, respectively [30, 31]. In this study, we compared the effect of GP and GO on surface modification of PCL electrospun scaffolds and their effect on cell compatibility and osteoinductivity to directly differentiate human umbilical cord Wharton’s Jelly-derived mesenchymal stem cells (hUCMSCs) into osteoblast like cells that are suitable for bone tissue engineering.

## Materials and methods

All the materials and reagents were acquired from Sigma Aldrich (MO, USA) and cell culture-related materials from Invitrogen (CA, USA) except where stated. Graphene (layer graphene sheets (GP) and Graphene Oxide (GO) (particle Size of 100–1000 nm) were kindly donated by Sachin Kochrekar, Department of Chemistry, Defense Institute of Advanced Technology, Girinagar, Pune, India.

### Preparation of scaffolds by electrospinning

PCL (10% w/v) was dissolved in Tetrahydrofuran and Methanol solvent in a 3:1 ratio for 30 h of magnetic stirring. Electrospinning was used to fabricate PCL scaffolds. Electrospinning parameters were flow rate of 0.8 mL/h, voltage of 12kv and a distance of 12.5 cm between the tip of the syringe and collector. The scaffolds surface of these electrospun PCL were modified by layer-by-layer deposition.

### Surface modification of scaffolds using layer-by-layer method

1 mg/mL GP or GO was dispersed in distilled water through sonication. PCL-GP or PCL-GO scaffolds were prepared by simply dipping the PCL scaffold repetitively in GP or GO solution for 2 min, followed by air drying. As previously shown, such intermittent 60 dip and dry cycles allowed optimum and uniform deposition. The preparation and characterisation of PCL-GP and PCL-GO scaffolds data were previously published [30, 31].

### Characterization of scaffolds

The surface deposition of GP or GO, the morphology of the scaffolds and their fiber diameter were examined by field emission scanning electron microscope (FESEM, Carl Zeiss, Germany) at an accelerating voltage of 15 kV. Scaffolds were cut into 5 × 5 mm squares, mounted on to sample stubs and sputter-coated with gold using SC 7640 sputter coater (Quorum Technologies Ltd, UK). The fiber diameter of the scaffolds was measured using image analysis software (ImageJ, National Institutes of Health, Bethesda, USA). The surface morphology of scaffolds was analyzed using tapping mode by atomic force microscopy (AFM, Asylum Research). The small portion of scaffolds was cut and stuck on a glass slide using cellophane tape. AFM imaging was performed with a scan rate of 1.0 Hz and a scan area of 10 µm. Fourier Transform Infra-Red (FTIR) spectra were recorded for all scaffolds (FTIR, Brucker, Germany). The spectra were obtained with 30 scans per sample ranging from 3000 to 500 cm^−1^. The surface wettability of scaffolds was measured by a contact angle goniometer (KRUSS, Germany). The sessile drop method with drop shape image analysis and pure water was used to calculate the water contact angle. A universal tensile machine (STS 248, Star Testing Systems, India) was used to determine the tensile properties. Scaffolds were cut into cylindrical shapes (n = 3) and tested with a maximum loading capacity of 100 N and a 5 mm/min strain rate to obtain stress–strain curves to calculate yield strength and tensile strength [32–34].

### Cell attachment and proliferation study

The hUCMSCs were isolated and expanded as previously described [30, 31]. The scaffolds were washed with PBS three times and sterilized with ethylene oxide (EtO). The hUCMSCs (1.0 × 104 cells/mL) were seeded onto scaffolds and incubated at 37 °C, 5% CO2 for 1, 4 and 7 days to study cell attachment, cell viability and cell proliferation activity. Cells seeded onto tissue culture plate (TCP) were used as control. Cell-seeded scaffolds were fixed with 4% paraformaldehyde and analyzed for cellular attachment using FESEM and cell viability and proliferation by 3-(4, 5-dimethylthiazol-2yl)-2, 5-diphenyltetrazolium bromide** (**MTT assay). MTT (5 mg/mL; pH = 7.4) was prepared in DMEM and filter sterilized through a 0.2 µM filter into a sterile, light-protected container. A sufficient amount of MTT solution was added onto cells seeded scaffolds and incubated for 3 h at room temperature. Dimethyl sulfoxide was used to dissolve formazan crystals. The newly formed purple coloured formazan was measured at 570 nm with a reference of 650 nm using a plate reader spectrophotometer (Hitachi) [35].

### Osteoinductive study for osteoblastic differentiation of hUCMSCs: Alizarin Red S staining

In serum-free growth media, the hUCMSCs were seeded on scaffolds for 14 and 21 days. The control was kept as a tissue culture plate with osteoblastic differentiation media containing DMEM supplemented with ascorbic acid (50 µg/mL), β-glycerophosphate (5 mM), dexamethasone (1X10-7 M), and nonessential amino acids (1%). After 14 and 21 days of culture, mineralization was analyzed by staining with 2% Alizarin Red S stain (pH 4.2). After PBS wash, samples were observed for Ca++ minerals under an inverted phase-contrast microscope equipped with a digital camera. For the quantification of alizarin red S staining, scaffolds were incubated with 10% acetic acid for 30 min, followed by heating at 85 °C for 10 min, then cooled and centrifuged at 10,000 rpm for 15 min. The above solution was taken and neutralized with 10% ammonium hydroxide. This solution was used to quantify Alizarin Red S by measuring absorbance at 405 nm [36–38].

### Von Kossa staining

Von Kossa staining was used to analyze the extent of mineralization on the scaffolds. 10% formalin was used to fix the samples, and 5% silver nitrate (AgNO3) solution was used to stain samples and kept for 60 min under UV light. The samples were washed with PBS to remove the excess stain and visualized under an inverted phase-contrast microscope.

### Statistical analysis

Statistical data was presented as Mean ± standard deviation. Origin Pro 8.5 Software was used to plot the graphs. Statistical significance was evaluated using ANOVA with post hoc tests. *P* < 0.05 was considered significant.

## Results

### Surface modified scaffolds with improved properties were prepared by layer by layer deposition

**Fig. 1:**
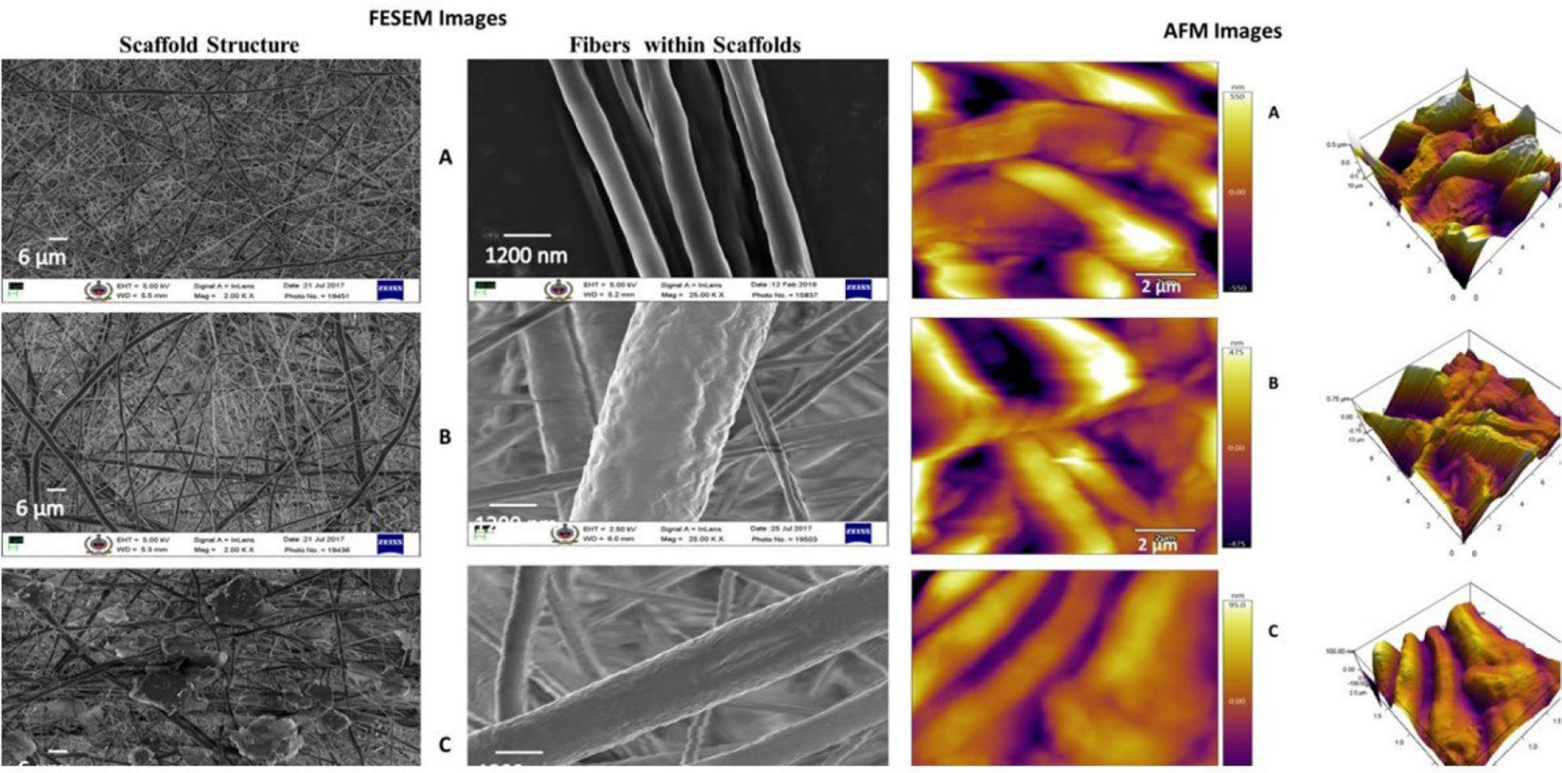
Field emission scanning electron microscopy (FESEM) shows the porous morphology of scaffolds and their respective fiber morphology. AFM images shows the surface roughness of scaffolds. **A** PCL; **B** PCL-GP; **C** PCL-GO (With permission, Kashte et al. [30, 31])

Electrospun plain PCL scaffolds and layer-by-layer surface-modified scaffolds were analyzed using FESEM (Fig. 1). There was a flat fiber appearance in the plain PCL scaffolds, whereas layer by layer surface-modified scaffolds with GP or GO presented rough fiber structures. There was a random but uniform distribution of GP or GO layers on PCL-GP or PCL-GO scaffolds. Fiber diameter (Table 1) was significantly increased in surface-modified PCL-GO scaffolds as compared with PCL-GP and plain PCL scaffolds.

**Table 1:** Properties of scaffolds fiber diameter, contact angle and mechanical properties were mentioned in the table (Data represented as Mean and standard deviation

S. No.	Type of Scaffolds	Fibre Diameter (nm) (Mean ± SD)	Contact angle (Mean ± SD)	Nature of Scaffolds	Tensile strength (MPa) (Mean ± SD)	Yield strength (MPa) (Mean ± SD)
1	PCL	226.4 ± 16.9	126.5 ± 0.3	Hydrophobic	0.85 ± 0.1	0.46 ± 0.01
2	PCL-GP	1029.5 ± 183.5	77.4 ± 0.3	Hydrophilic	0.86 ± 0.01	0.49 ± 0.01
3	PCL-GO	1929.6 ± 694.9	58.9 ± 0.5	Hydrophilic	1.21 ± 0.01	0.51 ± 0.02

There was no statistical significance between the groups) (With permission, Kashte et al. [30, 31])

Surface properties of plain PCL and surface modified PCL scaffolds were analyzed by atomic force microscopy (AFM) using tapping mode. Root mean square roughness (RMS) values were PCL (146 ± 10 nm), PCL-GP (270 ± 10 nm) and PCL-GO (222 ± 8 nm) (Fig. 1). The modified PCL-GP scaffolds showed the highest roughness, followed by PCL-GO compared to PCL.

**Fig. 2:**
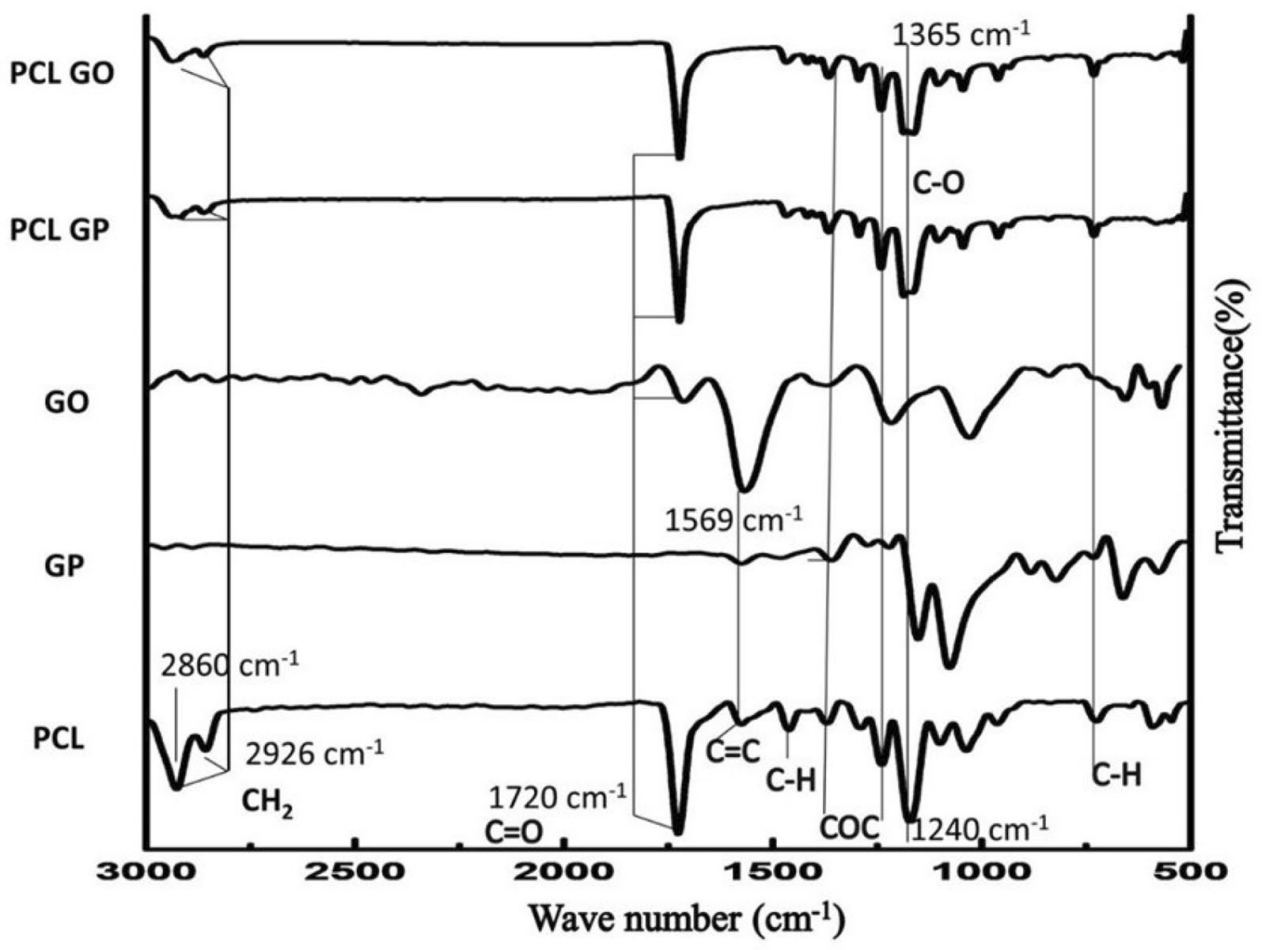
Fourier Transform Infra-Red spectroscopy (FTIR) spectra of the scaffolds (With permission, Kashte et al. [30, 31])

FTIR spectra (Fig. 2) showed the presence of PCL, GP or GO in the respective surface-modified scaffolds. The many overlapping peaks between PCL, GP, and GO make identifying them in surface-modified scaffolds difficult. However, integration and/or broadening of peaks confirmed the interaction of PCL with GP or GO in their respective scaffolds. The prominent peak of 1569 cm^−1^ and small peaks of GP between 2000 and 2500 cm^−1^ is reduced whereas 1365 cm^−1^ peak is broadened in PCL-GP and PCL-GO scaffolds.

Table 1 shows the water contact angles of scaffolds. PCL scaffolds were hydrophobic, while layer-by-layer modified PCL-GP, and PCL-GO scaffolds were hydrophilic. PCL-GO scaffolds showed the lowest water contact angle and highest hydrophilicity compared to PCL-GP and plain PCL scaffolds.

Table 1 shows the tensile strength and yield strength of scaffolds. GO showed a trend of enhanced tensile strength and yield strength of plain PCL scaffolds following surface modification, but there was no statistical significance.

### Cell compatibility studies

**Fig. 3:**
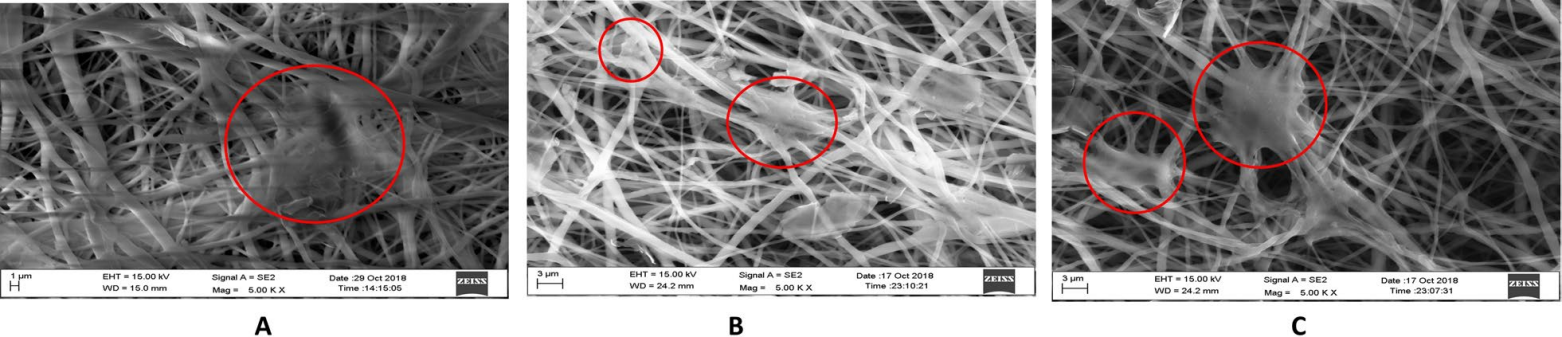
The FESEM images of cell attachment with Scaffolds. **A** PCL; **B** PCL-GP; **C** PCL-GO (Red circles shows the area of cell attachment) (With permission, Kashte et al. [30, 31])

There was better cell adhesion and cell proliferation on surface-modified scaffolds. An essential step in cell proliferation is the cell attachment and spread of cells on the scaffolds [39]. Although PCL, PCL-GP, and PCL-GO scaffolds showed hUCMSCs attachment on their surfaces after 24 h of culture, the hUCMSCs were well spread, fibroidal, and with evidence of greater adhesivity on PCL-GO scaffolds compared to the PCL-GP and PCL scaffolds (Fig. 3).

**Fig. 4:**
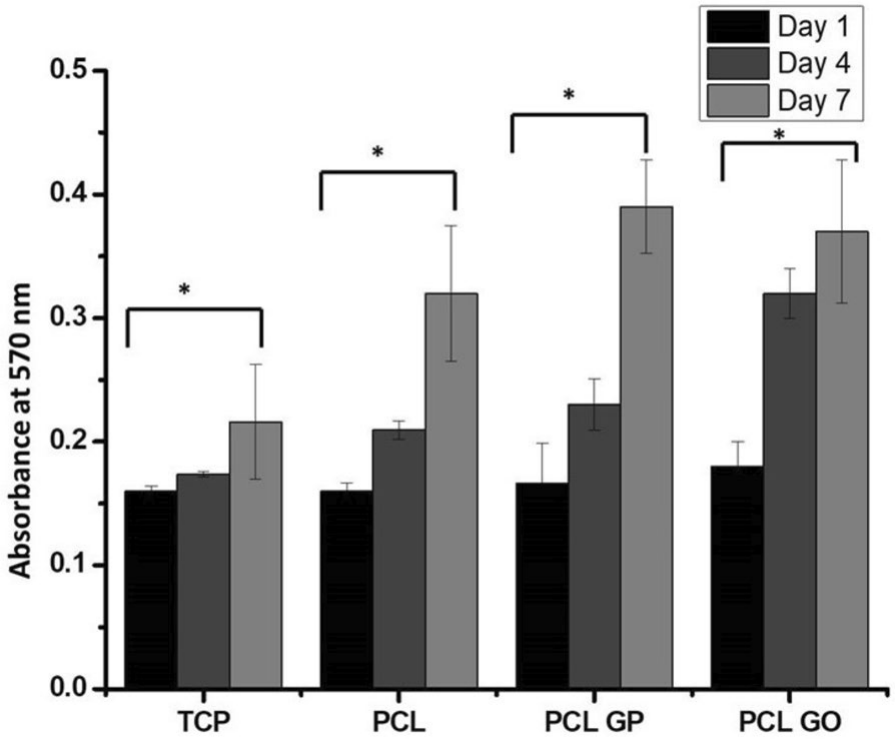
The cell viability and proliferation of hUCMSCs on the scaffolds for 1, 4, and 7 days of the culture were studied with an MTT assay (Data represented as Mean and standard deviation; *P < 0.05 for Day 1 and Day 7 in all groups. There was no statistical significance between groups) (With permission, Kashte et al. [30, 31])

Figure 4 shows the proliferation of hUCMSCs on different scaffolds as evaluated by the MTT assay. There was an increase in cell proliferation from day 1 to day 7 for all scaffolds. PCL-GP and PCL-GO scaffolds evidenced a trend for higher cell proliferation on day 7, but there was no statistically significant difference.

### Osteoinductive surface modified scaffolds showed osteoblastic differentiation of hUCMSCs

**Fig. 5:**
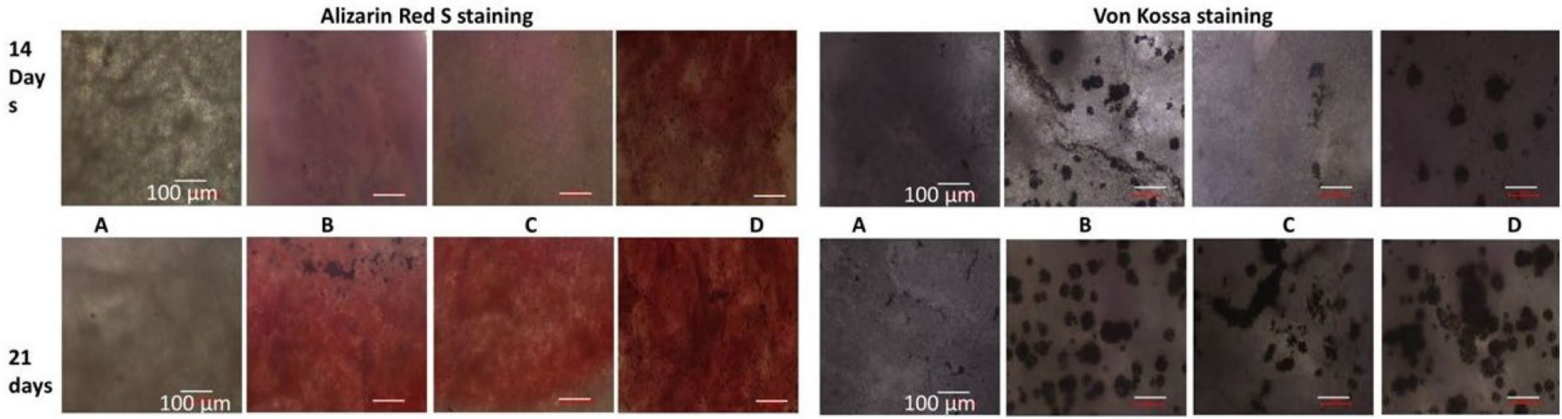
Alizarin Red S staining and Von Kossa staining of layer by layer scaffolds after 14 days and 21 days of differentiation of HUCMSCs. **A** PCL; **B** PCL-GP; **C** PCL-GO; (A–C: without Osteoblastic differentiation medium); **D** Tissue culture plate with Osteoblastic differentiation medium (With permission, Kashte et al. [30, 31])

**Fig. 6:**
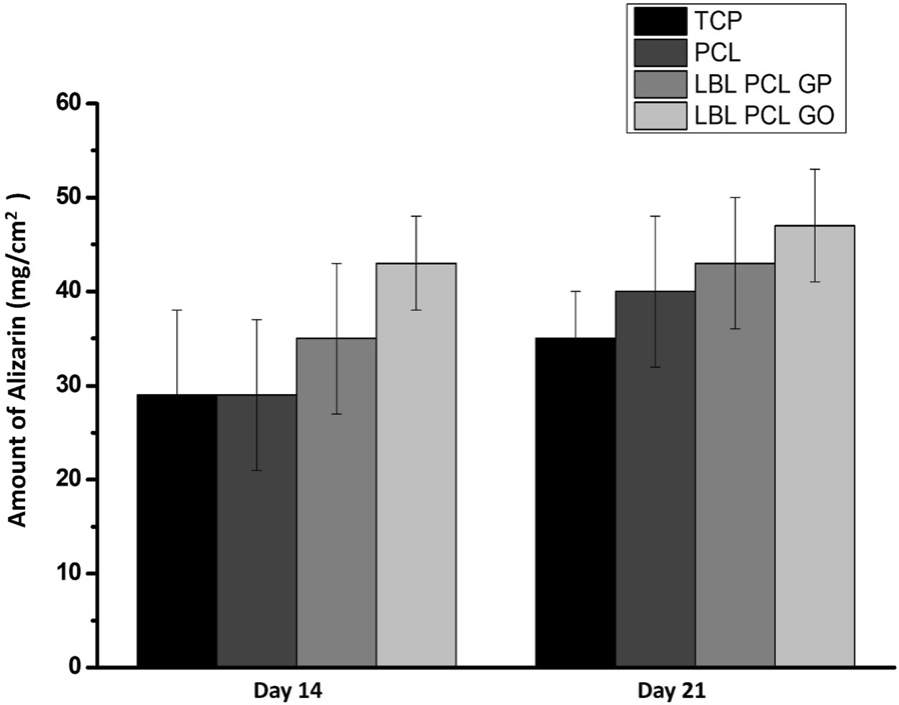
Alizarin Red S staining quantification of layer by layer scaffolds after 14 days and 21 days of differentiation of hUCMSCs (There was no statistical significance between groups) (With permission, Kashte et al. [30, 31])

Alizarin Red S staining was used to evaluate the mineralized matrix deposited by differentiated osteoblast-like cells (Fig. 5). After 14 days of culture of hUCMSCs onto PCL-GP and PCL-GO scaffolds, cells started to differentiate into osteoblasts. There was a trend for higher mineralization on PCL-GO scaffolds on the 21st day and 14th day compared to PCL-GP scaffolds, but there was no statistical significance (Fig. 6).

Also, Von Kossa staining, used to analyse the matrix mineralisation by differentiated cells (Fig. 5), showed the appearance of black precipitates from 14 days onwards. The black precipitates were distinctive, expansive, and more frequent on PCL-GO than PCL-GP and plain PCL scaffolds after 21 days of culture.

## Discussion

We evaluated the osteoinductive properties of layer-by-layer surface-modified PCL scaffolds with GP and GO for bone tissue engineering. Among the physiological properties, fibre diameter (Table 1) increased significantly in surface-modified PCL-GO scaffolds compared to PCL-GP and plain PCL scaffolds. This could result from greater interactions of GO compared to GP on the electrospun random fibres. Similarly, when immobilised with carboxy methylcellulose and laponite, electrospun PCL scaffolds showed an increase in the average fibre diameter of immobilised PCL-carboxy methylcellulose-laponite scaffolds [40]. Higher fibre diameter in the scaffolds facilitates migration and penetration of cells [41, 42]**.**

The modified PCL-GP scaffolds showed the highest roughness, followed by PCL-GO compared to PCL. This could result from the inherent roughness and undulating surface in the form of wrinkles of GP [25]. When PLA sheets were coated with polyethyleneimine-GP or polyethyleneimine-GO, the topography of PLA changed to rough mountain-like as compared to uncoated PLA films [21]. Also, GP or GO films on Si/SiO2 showed an increased presence of nanoripples [43]. Surface roughness increases the bioactivity of composites, hydrophilicity, and cytocompatibility [44]. Also, protein adhesion (fibronectin and albumin), cell adhesion, and cell proliferation improved on the rough surfaces [32, 39], which also promoted osteoblast proliferation and differentiation through matrix synthesis [45].

The FTIR spectra show many overlapping peaks between PCL, GP, and GO, and make identifying them in surface-modified scaffolds difficult. However, integration and/or broadening of peaks confirmed the interaction of PCL with GP or GO in their respective scaffolds. Similar results were observed by other researchers [32, 33, 46].

PCL-GO scaffolds showed the lowest water contact angle and highest hydrophilicity compared to PCL-GP and plain PCL scaffolds. This could be a consequence of the nature of GO, which has carboxylic and hydroxyl groups and their interactions [34]. Also, GP has carbon atoms at the edges with high chemical reactivity, allowing them to promptly react with other materials [14]. The addition of GO onto Poly (3-hydroxybutyrate-co-4-hydroxybutyrate) [34], PLA [21, 47], poly(lactic-co-glycolic acid) (PLGA) [48] decreased their contact angle, making them less hydrophobic and more hydrophilic. There is enhanced cell attachment and cell proliferation on the hydrophilic surface from topographical clues compared to hydrophobic surfaces [40, 45]. Also, there is improved absorption of fibronectin on the hydrophilic surface, which is vital in osteoblast adhesion in vitro [45]*.*

PCL-GO showed the highest tensile strength and yield strength compared to PCL-GP and PCL. GO contributed more to improving mechanical properties than GP. Also, the higher fiber diameter in PCL-GO resulted in increased values of mechanical properties [49]. The process of bone formation such as endochondral ossification is stimulated by higher mechanical strength scaffolds [50]. Desirable orthopedic scaffolds maintain the structure in load-bearing tissues such as bones in vivo [17, 51], and could be used in orthopaedic devices to prevent sudden breakage [46]. Therefore, the desired scaffolds should have higher mechanical properties to withstand the strains imposed on native tissues [52, 53].

PCL-GO scaffolds showed better cell attachment than PCL-GP and PCL. This may be consequent to suitable surface roughness and wettability properties of the PCL-GO scaffolds. PCL-GO scaffolds were more hydrophilic, but their surface was less rough than PCL-GP scaffolds. This could lead to attracting a greater number of cells and for their proliferation. Similarly, human osteosarcoma cells adhered and spread, showing flat morphologies on PCL blended with HA composite scaffolds [54]. Human fetal osteoblast cells showed cuboidal osteoblast-like morphology with filopodia formation on PCL nanofibrous scaffolds [32]. MSCs seeded on the electrospun PCL scaffolds immobilized with carboxymethylcellulose and laponite showed elongated morphology and better cell attachment [40].

All the scaffolds, PCL, PCL-GO and PCL-GP scaffolds showed increased cell proliferation from day 1 to day 7. Similarly, when MC3T3 cells were seeded on the PCL-GP and PCL scaffolds for seven days, there was higher cell proliferation but no significant difference among PCL and PCL-GP scaffolds [46]. Similarly, higher MG 63 cell proliferation was increased on GO and graphene nanoplatelets containing PLA scaffolds [47] which is also supported by the work of Chakrapani et al. [54] and Nayak et al. [43]. MSCs seeded on electrospun PCL scaffolds immobilized with carboxy methylcellulose and laponite showed significantly higher cell viability than electrospun PCL scaffolds [40]. The negative charge of GO and its polarity allows for van der Waals forces and electrostatic forces to interact with the functional groups of the proteins. The adsorption of proteins onto the surface of materials increases cell attachment and proliferation [13]. MTT assay confirmed the cell compatibility of all PCL, PCL-GP and PCL-GO scaffolds.

Alizarin Red S staining and its quantification showed higher mean mineralization values on PCL-GO scaffolds on day 14 and 21 compared to PCL-GP scaffolds. It indicates that GO could enhance the expression of osteogenic differentiation markers and can stimulate calcium deposition effectively as compared to GP. MSCs seeded on the electrospun PCL scaffolds immobilized with carboxy methylcellulose and laponite showed significant osteogenic differentiation after 21 days in the absence of any osteogenic media. There was higher expression of ALP and osteonectin by the differentiated cells [40]. PLLA-scaffolds also showed mineralization with simulated body fluid (SBF) after 14 days of incubation with Alizarin Red S staining [33].GP was an alternative to BMP-2 for osteogenic differentiation of hMSCs in osteogenic media [43]. There is increased expression of osteocalcin when MSCs differentiate into osteoblasts. Osteocalcin is a key regulator in bone metabolic activities [32]. However, another study reported that GP has higher osteogenic differentiation potential than GO in the presence of osteogenic induction media, possibly from the higher absorbance of dexamethasone and β-glycerophosphate by GP [25]. The present study demonstrated the higher osteogenic potential of GO compared to GP in the absence of any osteogenic medium. GP or GO are believed to promote the expression of osteogenic markers such as alkaline phosphatases (ALP), osteocalcin, Runx2, etc., through mechanical stimulation and induce the differentiation of MSCs into the osteoblast-like cells. Also, there is the involvement of regulation of MAPK signaling pathway, BMP signaling pathway, Wnt/β-catenin pathway for the regulation of osteogenesis [25].

Von Kossa staining shows similar results as Alizarin Red S staining and confirms differentiation of hUCMSCs into osteoblasts. Similarly, a combination of BMP2, BMP6 and BMP9 showed matrix mineralization by MSCs after 21 days of culture [55]. Serum and human plasma enhanced the osteogenic differentiation of MSCs after 28 days of culture in osteogenic media [56]. Biphasic calcium phosphate and calcium phosphate with conditioned medium showed matrix mineralization by MSCs after 21 days of culture [57]. Also, matrix mineralization by MSCs [58] fetal rat calvariae (FRC) cells [59] with osteoblastic induction medium was observed from 14 days onwards.

Local cells, including resident stem cells, are involved in the maintenance and restoration of organ function under physiological conditions. However, following acute trauma or disease, the sudden requirement of new cells during the healing response may exceed the plasticity of the local cell populations. Also, the ability of the tissue resident stem cells to re-enter the cell cycle and to asymmetrically divide is limited, which eventually curbs the extent of self-renewal following major loss of cells in damaged tissue. When such a localized self-healing power is exhausted, the repair or regeneration of damaged tissue requires to stimulate the patient’s body’s localized self-healing power or to provide the new cells that can integrate into the host during the healing response [60]. During such scenarios, osteoinductive scaffolds could be a game changer, as they can stimulate the differentiation of localized stem cells or transplanted stem cells, autologous or allogenic, into osteoblasts and will repair or regenerate the bone tissue.

GO differs from GP because it forms a uniform and stable suspension in water, whereas GP tends to form aggregates. Uniform, stable suspension of GO infiltrates the porous scaffolds, thereby modifying the surface of pore wall. GO has more hydrophilic groups as compared to GP. It can therefore react better with other biomaterials and can improve the hydrophilicity or mechanical strength much better than GP. The physical, chemical and mechanical properties of GO enhance the proliferation and stimulate the Wnt- β-catenin pathway for differentiation of stem cells. These could be the reasons for the higher osteoinductive properties of GO as compared to GP. This unique characteristic of GO makes it the material of choice for bone tissue engineering applications [13, 57].

Compared to graphene, graphene oxide improved the hydrophilic and mechanical properties of surface-modified layer by layer scaffolds. A trend for higher mineralization was evident in graphene oxide, but this trend is statistically not significant. Given their osteoinductive properties, graphene and graphene oxide enhanced the spontaneous differentiation of hUCMSCs into osteoblasts without any osteogenic media or growth factors. Thus, graphene and graphene oxide shows great potential for in vivo bone tissue engineering and may be a material to further investigate in the field of bone tissue regeneration.

## Conclusion

A trend of higher mineralization was evident in Graphene oxide, however this trend is statistically not significant. Therefore, graphene and graphene oxide shows potential for bone regeneration and bone tissue engineering applications. Future in vivo studies and clinical trials are warranted to justify its ultimate clinical use.

## Data Availability

The dataset generated during the current study is contained within the manuscript**.**
